# Metastasis: crosstalk between tissue mechanics and tumour cell plasticity

**DOI:** 10.1038/s41416-020-01150-7

**Published:** 2020-11-18

**Authors:** Bircan Coban, Cecilia Bergonzini, Annelien J. M. Zweemer, Erik H. J. Danen

**Affiliations:** Leiden Academic Center for Drug Research, Leiden University, Leiden, The Netherlands

**Keywords:** Epithelial-mesenchymal transition, Metastasis, Cancer microenvironment, Collective cell migration

## Abstract

Despite the fact that different genetic programmes drive metastasis of solid tumours, the ultimate outcome is the same: tumour cells are empowered to pass a series of physical hurdles to escape the primary tumour and disseminate to other organs. Epithelial-to-mesenchymal transition (EMT) has been proposed to drive the detachment of individual cells from primary tumour masses and facilitate the subsequent establishment of metastases in distant organs. However, this concept has been challenged by observations from pathologists and from studies in animal models, in which partial and transient acquisition of mesenchymal traits is seen but tumour cells travel collectively rather than as individuals. In this review, we discuss how crosstalk between a hybrid E/M state and variations in the mechanical aspects of the tumour microenvironment can provide tumour cells with the plasticity required for strategies to navigate surrounding tissues en route to dissemination. Targeting such plasticity provides therapeutic opportunities to combat metastasis.

## Background

Metastasis is the major cause of mortality associated with solid tumours. Tumour cells escape from the primary tumour mass, move through surrounding tissues, enter the circulation, and colonise distant organs to form secondary tumours. During this process, tumour cells have to navigate mechanical hurdles consisting of various extracellular matrix (ECM) structures and layers of cells. Cross talk between intrinsic properties of the tumour cells and mechanical aspects of their surroundings drives cellular plasticity that enables tumour cells to make this journey.

The cells of solid tumours are typically surrounded by a dense fibrotic tissue composed of cellular and acellular elements—the tumour microenvironment (TME)—which plays an active role in the aggressive metastatic behaviour of cancer.^1,2^ The TME comprises cancer-associated fibroblasts (CAFs), blood vessels and lymphatic vessels, immune-inflammatory cells, and neuroendocrine and adipose cells, all of which are embedded in an ECM, a structural network that sustains and shapes the three-dimensional architecture of tissues and organs. Within the TME, tumour cells are subjected to chemical (cytokines, growth factors) and physical cues that originate from the cellular elements as well as from the ECM. Together, these cues impinge on cellular signalling cascades in tumour cells thereby promoting tumour development and metastasis.

**Fig. 1:**
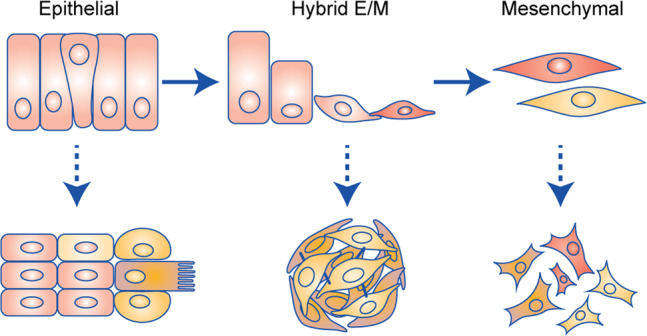
EMT regulates cell migration strategies. Upper row: During epithelial-to-mesenchymal transition (EMT), epithelial cells lose their tight intercellular junctions, form a transient hybrid E/M phenotype, and eventually lose their epithelial features while gaining mesenchymal features. This process is driven by a series of changes in gene transcription programmes. Lower row: migration strategies shift from collective migration, to migration with a high degree of plasticity, to individual migration as EMT progresses.

What triggers a cluster of tumour cells to transit to a motile state, crawl through surrounding tissues, and start the metastatic process? One concept is that this involves an epithelial-to-mesenchymal transition (EMT; Fig. 1), whereby epithelial cells lose their cell–cell contacts and apico–basal polarity, and acquire features of mesenchymal cells, allowing them to migrate and invade.^3^ This process is orchestrated by signalling molecules such as transforming growth factor (TGF)-β and Wnt, which induce downstream pathways that regulate a network of transcription factors to control the balance between key epithelial proteins (including mediators of cell–cell adhesion, such as E-cadherin and claudins) and mesenchymal proteins (such as vimentin).^3–5^ Transcription factors such as TWIST, SNAIL and ZEB induce EMT whereas GRHL2 and OVOL2 suppress EMT.^4,6,7^ EMT is important in embryonic development for cell migration and regulation of tissue differentiation and homoeostasis,^8,9^ but has also been associated with cancer initiation, development, and progression.^7,10,11^ However, the idea that a full transition from an epithelial to a mesenchymal state is required for metastasis has been challenged by observations from pathologists and studies using genetically modified mouse models.^12–14^

**Fig. 2:**
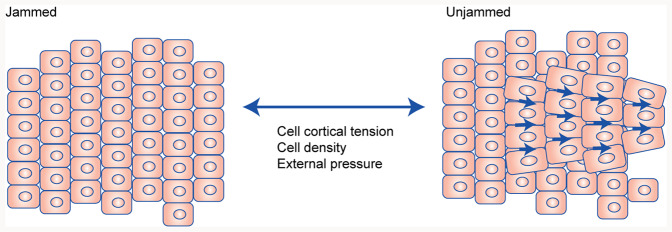
Unjamming transitions as an alternative means to trigger migration. Clusters of cells can switch between solid-like (jammed) and fluid-like (unjammed) states. In this case, changes in mechanical and geometric parameters in the tissue can trigger fluidisation (unjamming) in absence of the changes in gene transcription required for EMT.

An alternate concept explaining how groups of (cancer) cells may initiate movement is derived from active matter physics. It describes how changes in mechanical and geometric parameters such as extracellular pressure, cell density, and cortical tension, can trigger a shift from solid to fluid-like behaviour in cell clusters, without the need for transcriptional alterations such as those underlying EMT^15^ (Fig. 2). This shift is referred to as “unjamming” and transient shifts between jammed and unjammed states likely occur as tumour cell clusters navigate mechanical hurdles during the metastatic process. Notably, tumour cells are known to adopt a state referred to as partial EMT or a hybrid E/M state where epithelial and mesenchymal markers are combined. Crosstalk between mechanical aspects of the TME and the hybrid E/M state may drive plasticity and prime tumour cell clusters to unjamming, thereby allowing tumour cells to adapt to, and navigate physical hurdles and increase their metastatic potential.

Here, we focus on the early stage of the metastatic cascade where tumour cells leave the primary tumour, invade surrounding tissues, and enter the circulation. We present an overview of mechanical properties of the TME and discuss roles for (partial) EMT and unjamming in tumour cell migration strategies. We then explore bidirectional cross talk between the TME and partial EMT and discuss how this may contribute to plasticity and unjamming. While a detailed description of underlying molecular pathways is beyond the scope of this review, we discuss candidate therapeutic opportunities for targeting the TME and the hybrid E/M state to break crosstalk and plasticity in order to interfere with metastatic strategies.

## Mechanical aspects of the TME

Tumour cells are subjected to multifaceted physical cues within the TME.^2^ Increased stiffness and pressure, both solid and fluid, are the main macroscopic mechanical alterations that can be observed in the tumour bulk.

### Mechanical alterations within the TME

The components of the TME are not malignant per se—in fact, they are an important source of support for tissues in physiological conditions. However, as cancer progresses, many of these components are exploited by the tumour cells, causing a change in the mechanical properties of the TME. For example, CAFs can arise from resident fibroblasts and become activated in response to the release of growth factors such as TGF-β to acquire a tumour-promoting function.^2^ This process triggers a series of intercellular feedback loops: tumour cells recruit and activate stromal cells; these stromal cells contribute to the increased production and secretion of ECM, which, in turn, stimulates tumour progression. Ultimately, these events result in a stiffer TME, which confers increased resistance to physical deformation. This alteration in tissue tensional homoeostasis has been reported to enhance cancerous transformation.^16,17^ The dysregulation of ECM deposition, named desmoplasia, involves not only changes in terms of ECM quantity, but also its architecture and organisation.^18^ In particular, the main components of ECM that are dysregulated and associated with cancer progression are fibrillar collagens, fibronectin and hyaluronic acid (HA).^19^ These alterations in ECM contribute to the increased stiffness of the TME, which has been associated with increased malignancy and invasiveness in pancreatic ductal adenocarcinoma, breast cancer, colorectal cancer and prostate cancer.^20–24^

Besides alterations in stiffness, the mechanical TME is affected by increased solid and interstitial pressure as the tumour increases in size. ECM components such as HA and proteoglycans absorb water, which leads to an increase in solid pressure due to the resistance conferred by the surrounding tissue. In addition, proliferation of tumour cells generates solid pressure, as an increased uptake of soluble factors results in enhanced conversion into insoluble biomass.^25^ Expansion of the tumour bulk compresses tumour-associated blood and lymphatic vasculature, which, in turn, can affect the vascular integrity, ultimately leading to leaks and impaired drainage of lymphatic vessels. This impairment of the normal function of vessels leads to an increase in interstitial fluid pressure, which contributes to therapy resistance by inhibiting drug delivery to the tumour.^26^ In addition, impaired vascular integrity creates hypoxic regions, which induce activation of the transcription factor hypoxia-inducible factor (HIF)-1α, leading to tumour invasion and promotion of angiogenesis.^16,27^

### Active cellular mechanical remodelling of the TME

The physical alterations that occur within the tumour stroma are not just passive consequences of tumour growth. Tumour cells and CAFs actively change the mechanical properties of the TME through their interaction with the ECM. They adhere to ECM components through integrin receptors and use contractility mediated by the actin cytoskeleton and myosin motors to apply force onto these adhesions, causing cell-mediated deformation of the ECM proteins (termed strain stiffening), which contributes to the stiffening of tumour stroma.^25^ In a positive-feedback loop, the stiffer environment triggers an increase in actomyosin contractility and force application by tumour cells, causing further ECM stiffening.^28^ The tensile forces on the ECM also lead to the unmasking of new binding sites for integrins, further promoting cell–ECM interactions.^25,29^ In addition, tumour cells and CAFs remodel the ECM by enhancing collagen alignment through a process that requires contractility mediated by the GTPase Rho and its downstream effector Rho-associated kinase (ROCK), which has been associated with tumour invasion and attraction of vascular endothelial cells.^30–32^ Moreover, tumour cells can enhance crosslinking of collagen fibres in the ECM, which further augments stiffness of the tumour stroma. The main enzymes responsible for this crosslinking are tissue transglutaminase 2 and lysyl oxidases (LOXs), the expression of which is upregulated in several solid tumours. LOX enzymes, in particular LOX2, are upregulated in response to hypoxia and high levels of TGF-β, both of which are characteristic of the TME and associated with tumour progression and metastasis.^25,33,34^

The altered mechanical cues in the TME help to create a niche that supports tumour growth, invasion of surrounding tissues, and therapy evasion. Tumour cells sense the above-mentioned mechanical changes and transduce the mechanical input into intracellular biochemical signalling.^35^ A force-transmitting cytoskeleton is essential for cells to sense the mechanical properties of the environment and several signal transducers have been implicated in this process, including ion channels, cell matrix adhesion complexes and membrane-associated phospholipases. Within cell matrix adhesion complexes, mechanoresponsive elements including integrin receptors and associated cytoplasmic proteins such as focal adhesion kinase (FAK)^36^ couple the ECM to the cytoskeleton across the plasma membrane, providing mechanical homoeostasis between cells and the ECM.^37^ In conjunction with chemosensory signalling pathways (such as those activated by TGF-β and hypoxia mentioned earlier), this bidirectional signalling controls cell shape and migratory and invasive behaviour, as well as cell survival and proliferation.^38,39^

## Tumour cell migration: EMT and unjamming

Changes in the TME induce adaptive mechanisms, such as metabolic reprogramming in tumour cells, that, in addition to the intrinsic lack of homogeneity within tumours, contribute to the generation of tumour cell populations with diverse gene expression patterns and phenotypic features within a tumour mass.^40,41^ This ‘intra-tumour heterogeneity’ provides plasticity and confers a survival advantage on tumour cells to migrate, invade and reach distant organs.^42,43^ The conversion from a localised tumour into a full blown, disseminated cancer requires that tumour cells activate migration. EMT and unjamming represent two concepts explaining the acquisition of migratory capacity in tumours.

### EMT

EMT can contribute significantly to tumour heterogeneity and plasticity and has been proposed to drive the initiation of metastasis.^1,44,45^ For example, ErbB2 is a metastasis-promoting oncogene that is frequently overexpressed in non-invasive ductal carcinoma in situ. However, only a subset of ErbB2-overexpressing cells progressed to invasive breast cancer in animal models and patient tumours and in this subpopulation ErbB2 was accompanied by overexpression of 14-3-3ζ, which led to EMT.^46^ The notion that EMT represents a critical step for the initiation of metastasis is challenged by the lack of evidence for EMT in the histopathology of metastatic tumour tissues as well as in several studies using animal models.^12–14,47,48^ For example, depletion of the key EMT-promoting factors SNAIL or TWIST in a mouse model for pancreatic cancer or lineage-tracing using Fsp1 as an EMT marker in a mouse model for breast cancer failed to support a role for EMT in metastasis.^13,14,47^ On the other hand, a study using loss of E-cadherin as an EMT marker in a mouse model for breast cancer, associated the occurrence of spontaneous EMT in a small subpopulation of tumour cells with increased migration capacity.^48^ The interpretation of studies in favour of- and arguing against a critical role for EMT remains an ongoing debate.^11,49,50^ Importantly, defining EMT based on the expression of a single marker underestimates the dynamic nature of EMT as this process is likely to be a transient event in cancer.^51^ Moreover, EMT is a non-linear programme that can be defined and controlled by distinct gene networks in a cancer-type specific manner.^52,53^ It has been shown that a pro-metastatic effect of EMT depends not only on the final state but on the molecular route that leads tumour cells to that state.^54^ The reverse process, mesenchymal-to-epithelial transition (MET), occurs as tumour cells arrive at distant organs, and might be important for the formation of metastatic lesions, as disseminated tumour cells locked in a mesenchymal state fail to effectively colonise these organs.^48,55–57^

Notably, EMT also plays a role in other cell types in the TME including the generation of CAFs. CAFs can originate from normal resident tissue fibroblasts^58^ or mesenchymal stem cells.^59^ In addition, CAFs can arise from epithelial cells through EMT or from endothelial cells through endothelial-to-mesenchymal transition (EndMT) and both conversions are induced by TGF-β.^60,61^ It is largely unknown how these CAF populations differ in functionality, but they are all characterised by a myofibroblast phenotype that drives stiffening of the TME as described above.

### Partial EMT or hybrid E/M

Rather than a complete EMT, transient subtle changes in the balance between pro- and anti-EMT transcription factors that result in a partial EMT or ‘hybrid E/M’ state might be more relevant in the context of cancer (Fig. 1). Indeed, both epithelial and mesenchymal markers can be co-expressed in a single tumour cell in hybrid E/M and a range of intermediate states may exist.^62–65^ One advantage of maintaining an epithelial phenotype, such as expression of E-cadherin in a hybrid E/M state is an increased survival fitness through cell–cell contacts in tumour clusters in the circulation.^66^ Hybrid E/M is also associated with increased stemness, which, in turn, is linked to elevated plasticity and self-renewal capacities as compared with completely E or M states in breast cancer.^63,67,68^ Additionally, a tumour that harbours subpopulations of cells residing at different stages of a fluid, cancer-associated hybrid E/M state might have an optimal capacity to cope with variations in the TME and progress towards metastasis. A hybrid E/M state confers phenotypic and molecular diversity, which provides cellular plasticity, empowering tumour cells to navigate various physical hurdles during their journey to metastatic sites while maintaining expression of epithelial markers and intercellular adhesion.^3,7,63,64,69–72^ Indeed, in a mouse model for breast cancer, a hybrid E/M state induced the formation of tumour cell subpopulations with varying degrees of invasiveness and metastatic potential.^63^ The existence of hybrid E/M cell populations and their association with enhanced metastatic features including migration and intravasation, were corroborated by studies on ovarian and pancreatic cancers.^73,74^ A biophysical model also showed that hybrid E/M states give rise to heterogeneous clusters migrating collectively and leading to the circulating tumour cell clusters as observed in animal models and patients.^75^

### Unjamming transitions

The collective movement of cell clusters has also been studied using principles from active matter to describe transitions between arrested (“jammed”) and moving states (“unjammed”) in cell aggregates.^15^ In this case, changes in mechanical and geometric parameters in the tissue trigger fluidisation in absence of EMT^15^ (Fig. 2). In epithelial cells grown as a monolayer, introducing a wound or perturbing endocytosis induces unjamming and creates a transition from a static to a flowing state.^76–78^ Likewise, compressive stress mimicking a bronchospasm triggers a transition in a monolayer of airway epithelial cells from a solid-like jammed phase to a fluid-like unjammed phase.^79^ A solid-to-fluid transition is also observed during development in *Xenopus laevis*, in which a hybrid E/M is associated with a fluid, but still collective, state of migrating neural crest cells.^80^ A study using MCF10-derived tumouroids showed that a similar fluidisation process occurs at the edges of densely packed breast cancer cells.^81^

If and how the early steps of metastasis follow similar principles, represents an urgent, unresolved issue. In breast cancer, clusters of invading tumour cells are more prone than individual cells to survive. These clusters promote metastasis formation in mouse models and give rise to oligoclonal clusters in the circulation that are associated with poor prognosis in patients.^82,83^ Likewise, circulating tumour cell clusters can arise from collective cell migration and intravasation in renal cell carcinoma, lung cancer and invasive melanoma.^84–86^ Whether cluster invasion in the complete absence of a partial EMT fully explains these findings is unresolved. EMT-like changes have been detected in circulating tumour cells.^87^ Yet, clusters of circulating tumour cells are largely epithelial and evidence in favour of E/M hybrid clusters is still scarce, suggesting that unjamming of fully epithelial tumour tissues may occur.

Tumour cells in the centre of a tumour mass are likely to be jammed but increased pressure might drive a switch from a solid to a fluid-like state. Indeed, multiphoton microscopy in a spontaneous mouse model for intestinal cancer has shown coordinated migratory patterns in the tumour core that are indicative of a fluid-like behaviour.^88^ Such movement has been suggested to be critical for cell mixing inside the tumour, which allows the most aggressive clones to effectively replace all other cells.^89^ In the outer regions, tumour cells are prone to mechanical stress due to a high abundance of ECM, which results in further unjamming.^15^

### Collectivity in tumour cell migration strategies

Unjamming, as well as a hybrid E/M state, leads to a fluid-like migration of clusters of tumour cells that maintain cell–cell contacts. It has been reported that high expression of EMT-promoting transcription factors such as Snail and Twist leads to the collective migration of tumour cells that exhibit epithelial and incomplete mesenchymal features.^90,91^ Likewise, unjamming of breast cancer cells triggered by a cascade of growth factor receptor internalisation, activation of extracellular signal-regulated kinase/mitogen-activated protein kinase and cytoskeletal remodelling, induces collective migration.^81^ Glioma cells infiltrate the brain as multicellular networks and breaking cell–cell interactions by downregulating p120-catenin was found to decrease infiltration capacity, again indicating that the ability to maintain cell–cell contacts is important.^92^ It is likely that the interaction between molecular programmes induced by hybrid E/M and local, physical cues in the TME creates routes for subpopulations of tumour cells to unjam and start disseminating.^46,93^

**Fig. 3:**
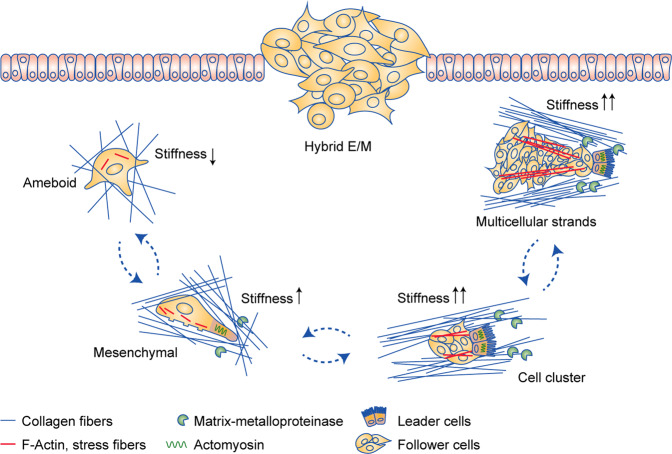
The hybrid E/M state provides plasticity and the local TME dictates collective and individual migration strategies. In a low stiffness environment, hybrid E/M cells migrate individually through ECM networks in an amoeboid or mesenchymal fashion. Amoeboid cells move through existing openings in a soft ECM of high porosity using few ECM adhesions and stress fibres, independent of protease activity. Mesenchymal migration in regions of somewhat higher stiffness and lower porosity is accompanied by increased formation of ECM adhesions, stress fibres and actomyosin contractility, and requires protease activity (mediated for instance by matrix metalloproteases (MMPs)) to generate openings through which to migrate. A further increase in TME stiffness promotes collective migration of hybrid E/M cells. Collective migration can take the shape of cell clusters or multicellular strands and involves contractile and proteolytically active leader cells creating the path for follower cells. Collectively migrating cells can make use of pre-existing large-scale mechanical structures in the TME such as channels or interphases between cell layers. Interconversion between the different migration strategies is dictated by local variations in the mechanical aspects of the TME, and the hybrid E/M state provides tumour cells with enhanced plasticity to respond to such cues.

Mixed individual and collective migration modes are observed in tumours of distinct origin: even mesenchymal tumours such as sarcomas switch from an individual to a collective migration mode in areas of particularly dense ECM structures.^94^ Single cells can move through ECM networks by adopting amoeboid or spindle-like mesenchymal shapes:^95^ amoeboid cells generate few ECM adhesions and stress fibres whereas mesenchymal migration is associated with strong ECM interaction and actomyosin contractility.^93^ Collectively migrating cells adopt different morphologies such as sheets, strands, multicellular tubes and masses with irregular forms (Fig. 3).^96^ Inside groups of collectively migrating cells, intercellular junctions can sense and integrate chemical and mechanical cues from the environment. Migrating clusters are usually organised into two cellular populations: leader and follower cells. The leader cells are responsible for sensing the microenvironment and generating traction forces to move the remainder of the group, which they do by proteolytically remodelling the matrix in order to create a path through which the collective group can navigate.^97^ It has been suggested that a collective migration strategy might be thermodynamically favourable by alternating leader cells that are exposed to a long-range strain field at the invasive front.^98^ In vitro models also showed how switching leader and follower positions, enables groups of breast cancer cells to invade through areas of high ECM density.^99^

## Crosstalk between partial EMT and TME mechanics

Plasticity of tumour cells allows them to switch between distinct modes of migration, which provides them with the means to navigate the mechanical complexity of their environment.^90^ A transition between escaping individual cells and regrouping collectives can be observed in collective strands of invasive cells.^100^ The hybrid E/M state probably supports such plasticity and the local physical properties of the TME can determine the level of individualisation. Indeed, using theoretical, in vitro and in vivo models shows how a weakening of cell–cell adhesion (as occurs in hybrid E/M) cooperates with ECM confinement to drive unjamming, fluidisation and, ultimately, cell individualisation.^101^ Thus, the interaction between molecular features of tumour cells and local properties of the TME can drive metastasis by mediating interconversions between collective and individual behaviour (Fig. 3).

### TME stiffening promotes EMT

**Fig. 4:**
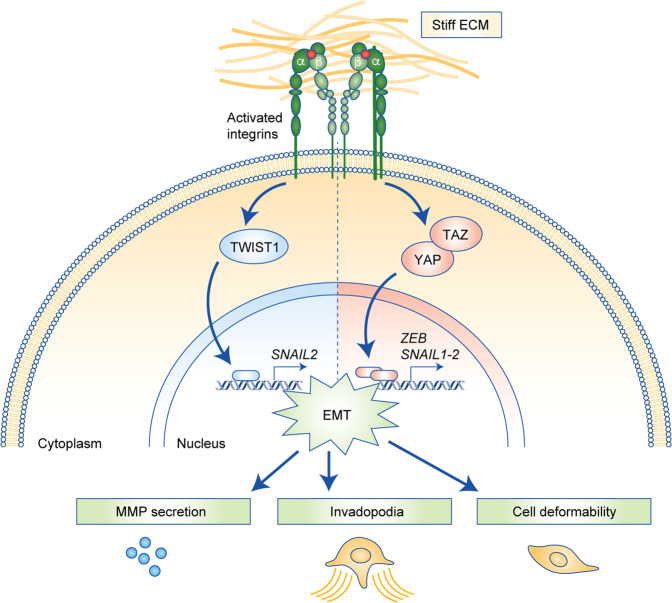
Mechanotransduction drives EMT in response to mechanical cues from the TME. An increased stiffness in the TME is sensed by integrins, which activate downstream intracellular signalling, ultimately resulting in the nuclear translocation of EMT-associated transcription factors and transcriptional co-activators, such as TWIST and YAP/TAZ. In the nucleus, these factors will bind to and regulate the transcription of target genes such as SNAIL and ZEB, causing a shift between epithelial (E) and mesenchymal (M) features. As tumour cells undergo EMT, cell deformability, proteolytic activity and the formation of invadopodia increase, driving enhanced migratory and invasive capacity.

Tumour cells sense and respond to mechanical stimuli from the TME.^35,39^ Integrins and associated intracellular proteins bidirectionally transmit force between the ECM and the cytoskeletal network and associated molecular motors (e.g. myosins), which facilitates ECM remodelling and regulates canonical signal transduction pathways that control cell fate.^102^ Mechanical cues from the TME, such as increased ECM density and stiffness, can stimulate EMT^20,103–106^ and act in concert with soluble EMT-stimulating factors, such as TGF-β.^103,107,108^ Important mediators of mechanically-induced EMT are the transcription factors TWIST1 and YAP/TAZ,^109,110^ which, upon matrix stiffening and subsequent intracellular transduction of mechanical signals, are induced to translocate to the nucleus to influence the expression of several genes that promote EMT (Fig. 4).^103,110–112^ A positive feedback loop is also generated by the interaction with HA in the TME. The interaction between CD44 on the cell surface and HA in the ECM induces the activation of ZEB1, which, in addition to promoting EMT also inhibits epithelial splicing regulatory protein 1 (ESRP1) leading to the up-regulation of hyaluronic acid synthase 2 (HAS2) and increased HA production.^113^ Thus, the chemical composition and stiffening of the TME can promote (partial) EMT in tumour cells. Notably, cells appear to possess a “mechanical memory” i.e., prolonged exposure to a stiff ECM causes EMT-like behaviour with nuclear localisation of YAP, high actomyosin contractility, and large cell matrix adhesions and this phenotype is maintained when the cells move to a soft environment for as long as the factors mediating the mechanical memory suppress a transcriptional switch.^113–115^

### EMT and tumour cell mechanics

Whereas stiffening of the TME drives EMT and the aggressive behaviour of tumours,^116^ tumour cells themselves have been observed to be “more deformable” or “softer”.^117^ EMT might play a role in such softening of tumour cells. Cells undergoing EMT change their morphology, lose adhesive properties and undergo actin cytoskeletal rearrangement, which all influence cell stiffness and tension with neighbouring cells and the ECM.^118^ Mesenchymal-like cells tend to reduce their stiffness and become softer in response to force application, while epithelial cells are more likely to stiffen in response to the same force application.^119^ Accordingly, EMT-promoting transcription factors such as SNAIL and TWIST1 promote increased cellular deformability,^120^ which facilitates migration through ECM networks and intravasation.^119^

Actin fibres connect integrin-containing adhesions with the nuclear envelope through the linker of nucleoskeleton and cytoskeleton (LINC) complex, thereby creating a physical connection between the ECM and the nucleus.^121^ This interaction is important for tuning the mechanical properties of the nucleus during migration in confined spaces. Indeed, nuclear deformability is a rate-limiting step for cell migration and some level of nuclear rupture has been observed during the migration of tumour cells in a confined space.^122–124^ The nucleoskeletal lamins regulate stiffness of the nuclear envelope and thereby determine a cell’s migratory capacity in confinement.^125^ How (partial) EMT affects nuclear mechanics remains to be elucidated but a hybrid E/M will increase cellular and, perhaps, nuclear deformability to increase plasticity, allowing tumour cells to adapt to confinement and enhance migratory potential.

### EMT and tumour cell-mediated modulation of the TME

As tumour cells undergo EMT, they also increase the production of soluble proteases or membrane-anchored MMPs, which allows invading tumour cells or tumour cell clusters to remove barriers or create tracks.^29,126,127^ The number of invadopodia—specialised actin-based membrane protrusions in which localised proteolytic activity degrades ECM—is also increased in tumour cells that are subjected to a stiffer environment or dense fibrillar collagen structures.^128,129^ Likewise, EMT induced by transcription factors including TWIST1 and ZEB1, promotes the formation of invadopodia in tumour cells.^130,131^ Thus, the interconnection between stiffening of the TME and EMT discussed above might enhance the ability of tumour cells and tumour-cell clusters to proteolytically degrade the ECM and break through tissue barriers. The importance of proteolytic ECM degradation, however, depends on the migratory strategy. While enzymatic breakdown of ECM is necessary for collective migration, individually migrating cells can either proteolytically remodel their surrounding ECM or adapt their shape to the already existing gaps.^93^ EMT driven by ZEB1 also leads to increased expression of LOXL2,^132^ which not only causes enhanced collagen crosslinking and TME stiffening but has been found to stimulate an EMT-associated transcription network,^133^ providing yet another positive feedback loop between EMT and the TME.

## Targeting the TME and hybrid E/M state

Interfering with the metastatic process remains a major challenge. Crosstalk between tumour cells and the TME is complex and dynamic and provides plasticity that allows tumour cells to adapt to different environments and escape therapy. We have discussed the mechanical interplay between the TME and tumour cells and a role for partial EMT in this process. Several candidate targets exist, which, when inhibited, might block this mechanical interaction and prevent tumour cell plasticity, including integrins,^134,135^ vimentin,^136^ Rho/ROCK and actomyosin contractility^137^ and FAK.^36,134,135,138^ Notably, however, interfering with tumour–TME interactions can also have unexpected and undesirable effects. For example, whereas inhibition of FAK in a mouse model for pancreatic cancer attenuated the cancer-promoting activity of the fibrotic stroma, limited tumour progression and enhanced survival,^138^ depletion of CAFs, which might be expected to have a similar effect, actually led to more aggressive tumours and reduced survival.^139^ One explanation is the heterogeneity of CAFs in pancreatic and other cancers that may have diverse impacts on tumour growth and progression within the TME, including immune-modulation.^140,141^

Strategies that simultaneously target different mechanisms of tumour cell plasticity, including the hybrid E/M state, might prevent tumour cells from adapting to changes in the TME.^138,142^ A network topology-based modelling approach has been applied to identify approaches for interfering with feedback loops in EMT networks, which may point to new strategies to interfere with plasticity and, hence with metastasis.^143^ Signal transduction cascades and transcription factors promoting a stable hybrid E/M state might serve as promising therapeutic targets, including GRHL2, OVOL2, NUMB and NRF2.^75,144,145^ Such a strategy has been successfully explored in breast cancer cells, in which the expression of SNAIL is associated with the hybrid E/M state. Deletion of SNAIL or either deletion or overexpression of ZEB1 pushed cells either in a complete E or in an M state, in each case resulting in attenuated capacity to form tumours.^146^ Despite these promising results, strategies that drive hybrid E/M cells into MET pose the risk of driving metastatic outgrowth of already disseminated tumour cells.^48,55–57^ On the other hand, strategies that lock cells in the M state might attenuate the outgrowth of primary and secondary tumours but drive the dissemination of individual tumour cells.^56^ An alternative promising strategy that exploits the highly plastic hybrid E/M state has made use of a combination of peroxisome proliferator-activated receptor γ (PPARγ) activation and MEK inhibition to enforce transdifferentiation of the tumour cells into post-mitotic adipocytes.^147^ This points to an exciting possibility that while plasticity allows tumour cells to adapt to different environments during metastasis it also represents a state that is vulnerable to differentiation therapy.

## Conclusions

In this review, we have discussed the dynamic interactions of tumour cells with the TME. In particular, we highlighted the importance of tissue mechanics and the role of (partial) EMT in the early steps of the metastatic cascade. The TME provides a pathological mechanical environment that tumour cells sense and respond to. The initiation of the metastatic cascade requires acquisition of a migratory phenotype that is influenced by this environment. The role of EMT in this process is likely different in different tumour types and in most cases involves a partial EMT or hybrid E/M state. EMT and unjamming provide distinct mechanisms to initiate movement and to what extent hybrid E/M sets the stage for unjamming of epithelial tumour cell clusters is poorly understood. The hybrid E/M state provides tumour cells with plasticity affecting stemness, tumour growth, and migration, allowing them to navigate variations in the mechanical TME as they use collective strategies to invade local surrounding tissues and enter the circulation. It is the bidirectional cross talk between partial EMT-driving molecular programmes in the tumour cells and the heterogeneous local mechanical properties of the environment that drive the early stages of the metastatic cascade. Further insight into the dynamic nature of this process at different stages of the metastatic cascade is required. This will depend on integration of multiscale theoretical models, in vitro models incorporating tumour heterogeneity and relevant mechanical variations in the TME, and in vivo models that capture the full complexity of the metastatic process. Disrupting mechanical tumour–TME interactions and/or tumour plasticity at the level of the hybrid E/M state offers promising avenues for therapeutic strategies. In this area, we have only just begun to scratch the surface of what might be possible.

## Data Availability

Not applicable.
